# Understanding ESL Teachers’ Agency in Their Early Years of Professional Development: A Three-Layered Triadic Reciprocity Framework

**DOI:** 10.3389/fpsyg.2021.739271

**Published:** 2021-09-08

**Authors:** Jing Huang, Jesse W. C. Yip

**Affiliations:** ^1^Department of Education Studies, Hong Kong Baptist University, Hong Kong, SAR China; ^2^School of Humanities and Languages, Caritas Institute of Higher Education, Hong Kong, SAR China

**Keywords:** Hong Kong secondary English as a second language teachers, teacher education, teacher development, teacher agency, teacher identity

## Abstract

Drawing upon the Triadic Reciprocity Framework, this longitudinal qualitative multiple-case study examined how three Hong Kong secondary English as a second language (ESL) teachers exercised their teacher agency to take control of their teaching and professional development. More specifically, the study aimed at exploring how teachers’ intentions and actions for the establishment of their professional identity were afforded and constrained by their workplaces. Findings reveal that these ESL teachers exercised different degrees of proactive, reactive, and passive agency. The four properties of human agency, i.e., intentionality, forethought, self-reactiveness, and self-reflectiveness, influenced the teachers’ proactive, reactive, and passive agency when they responded to personal, behavioral, and environmental determinants. The findings shed light on a three-layered Triadic Reciprocity framework on teacher agency and contributes to a systematic and comprehensive discussion about the various internal and external factors that might exert influences on agency of early career teachers. This study offers pedagogical implications for school teachers, school leaders, and policy makers in Hong Kong and beyond.

## Introduction

Confronted with heavy workload, lower positions, and high social responsibilities, language teachers at their early career stage are likely to undergo identity struggles and professional burnout. Understanding of teachers’ professional development and practice in contexts is crucial for providing responsive support for them. Recent literature on second language teacher education has highly appreciated the concept of agency, as it “critically shapes their [teachers’] responses to problematic situations” (Biesta and Tedder, 2006, p. 11). It also echoes the sociocultural approach in second language teacher education, in which teachers are no longer treated as knowledge transmitters (Johnson, 2009). Therefore, it is significant to investigate how teachers position themselves in classroom teaching and how their professional agency is shaped by the specific contexts (Vongalis-Macrow, 2007).

Language teacher agency is a relatively under-explored area in teacher education (White, 2018). An understanding of teacher agency can shed light on teacher effectiveness, expertise, and attitude during the initial years of teaching. The experiences that teachers obtain during the early career stage affect their actions, efficacy, and retention in professional development. In recent years, a large number of teachers quitted teaching within a few years after graduating from a teacher-education program (Shakrani, 2008; Fantilli and McDougall, 2009). This trend has become even more serious as rates of teacher attrition and turnover are consistently higher than those of other professions (Ingersoll and Strong, 2011). Corcoran (1981, p. 19) attributed this phenomenon to the “transition shock” of teachers when they face contradiction between the ideal and actual realities of teaching.

This study explored how three English as a second language (ESL) teachers exercised their agency to take control over their teaching during their early in-service years. Agency is dependent on a combination of personal, behavioral, and environmental determinants. To explore teacher agency in secondary school contexts, this study employs the Triadic Reciprocity Framework Core Agency Concepts (TRFCAC) model (Jenkins, 2020) to examine the complexities and obstacles teachers encountered. The study reveals the multiple-layered nature of teacher agency to inform our knowledge of teacher development. A close scrutiny of teacher agency provides valuable implications for teacher education and development. It helps understand how early teachers respond to challenges in their teaching and how their pedagogical intentions and actions may be afforded and/or constrained by the environment.

## Teacher Agency

Agency is often conflated with action and is often framed in opposition to structure, i.e., factors that influence or limit the available choices or capacity to be agentic (Priestley et al., 2016). Agency thus refers to the capacity to “act otherwise” (Giddens, 1984, p. 9), the “socioculturally mediated capacity to act” (Ahearn, 2001, p. 112), or “action potential, mediated by social, interactional, cultural, institutional, and other contextual factors” (van Lier, 2008, p. 171). Agency, including learner and teacher agency, “entails actions, and often suggests actions that arises from deliberation and choice” (Allison and Huang, 2005; Huang, 2011, p. 230; Huang, 2013). Such a conceptualization of learner and teacher agency enables a close scrutiny of learners’ and teachers’ responses to the constraints and affordances in a particular socio-institutional context, which in turn offers a useful way to problematize and look critically at actual learning and teaching situations. According to Bandura (2001, p. 8), an agent has to be a planner, forethinker, motivator, and self-regulator, for which agency involves “not only the deliberate ability to make choices and action plans, but the ability to give shape to appropriate courses of action and to motivate and regulate their execution”. Bandura’s (2001) argument may explain why ordinary classroom teachers can also achieve agency and become “agents of change” if they are situated in a supportive environment (Priestley, 2011; Priestley et al., 2012, 2013).

Studies in delineating professional teacher agency has reported that teacher agency, or teachers’ ability to act toward teaching requirements, are inseparable from the contexts in which they operate (Lasky, 2005). Such description is reasonable, as teachers are the actors who act “by-means-of-an-environment rather than simply in an environment” (Biesta and Tedder, 2006, p. 19). Teachers’ adoption of actions is a coping response to deal with their working environment (Priestley et al., 2016). We may assume a reciprocal relationship between the teachers and the contexts. Priestley et al. (2015) formulated an ecological model comprising three dimensions (see also Biesta et al., 2015; Priestley et al., 2016): iterational (i.e., teacher agency arises from teachers’ accumulated teacher experiences and previous patterns of thoughts and actions), practical-evaluative (i.e., teachers can make practical judgements based on evolving situation), and projective (i.e., teachers can be motivated by an intentional act of creating a future).

From a sociocultural perspective, teacher agency entails a teacher’s conscious efforts to “resist feelings of powerlessness and negativity experienced as a by-product of the environmental conditions” (Ollerhead, 2010, p. 607). In dealing with the fluid “constraints and affordances” (White, 2018, p. 197), teacher agency is dynamic rather than static (Imants and Van der Wal, 2020). Teacher agency manifests in three ways: proactive agency, reactive agency, and passive agency (Jenkins, 2020). Teachers adopt proactive agency when they plan for and initiate any changes as a personal choice. Teachers exercise reactive agency when they are required to make changes as a result of an environmental influence, e.g., directive influence from the leadership. Teachers enact passive agency when they are obligated to succumb to the school leadership and resist required changes.

Hence, teacher agency is not an attribute residing in individual teachers, but rather something achieved through engagement with specific contexts (Biesta et al., 2015; Priestley et al., 2016). In response to pedagogical challenges in specific contexts, while some teachers can be “reflexive and creative, acting counter to societal constraints,” some can be “enabled and constrained by their social and material environments” (Priestley et al., 2013, p. 189).

## Empirical Studies on Teachers’ Professional Experiences and Agency

Beginning teachers often encounter problems in exercising agency for professional development. Orr (2012) applied the Marxist concept of alienation to analyze trainee teachers’ experiences in coping with difficult circumstances, while learning to teach. The lack of control was found in trainee teachers’ accounts. Orr (2012, p. 61) described the teachers’ vulnerability to learn how to cope with loss of control as a type of accepting alienation. When addressing difficult circumstances, trainee teachers found themselves in “a double bind of alienation” while seeking to “escape society’s perceived ills.” However, teachers demonstrated different agency, even when they were working in the same institution. Ollerhead (2010, p. 616) investigated two ESL teachers’ professional journeys in the Australian context. One teacher contested the traditionally powerful policy conditions at a micro level through her own creative and intuitive classroom teaching experiences. Another teacher strictly adhered to and comply with the policies. Such adherence resulted in low student participation and insufficient motivation in teaching. The two teachers’ experiences draw attention to “the potential for the powerful and transformative effects of teacher agency.”

Harfitt (2015, p. 33) investigated teacher attrition and retention through the lens of two Hong Kong novice teachers’ narrative accounts (i.e., their stories to live by). The study documented how teachers’ personal and professional landscapes “bumped” and “merged,”, shedding light on the possibility “to see identity change as a framework for conceptualizing the global phenomenon of teacher attrition” (p. 33). Findings of the study pointed to the necessity of allowing teachers to have adequate time to develop themselves as teachers instead of expecting them to cope with the work and tasks that are normally reserved for more experienced teachers (see also Orr, 2012).

Trent (2019) examined why some graduating teachers in Hong Kong chose not to teach. A repeated theme in his study is the participants’ perception of being positioned in ways that deny their agency in their efforts to construct their desired professional identities. The blockage of their agency led to their vulnerability in pursuing a teaching career. Trent (2017, p. 102) focused on Hong Kong early career English teachers’ agency and the reasons for leaving the teaching profession. A major reason was the “conflict between the discourse of teaching as an individual accomplishment and the discourse of teaching as a community accomplishment to fill the identity ‘teacher’ with different meanings.” The teacher participants found it difficult to align teacher identity with agents of changes, and thus become unable to change certain teaching practices and activities. Both studies by Trent (2017, 2019) highlighted the importance of agency in teacher development.

Given the crucial role of agency in teachers’ professional development, there were also studies specifically focusing on teacher’ agency development. For example, in a study focusing on Chinese language teachers in international schools in Hong Kong (Lai et al., 2016), the findings reveal that the teachers’ exercise of professional agency is subject to the influence of social suggestions, power relations, teachers’ professional and social positioning, and the imposed identity and social roles in the school contexts. Drawing upon data from four Korean teachers, a study by Hiver and Whitehead (2018, p. 77) demonstrates that teacher agency is a complex and interrelated system of components. In particular, the four teachers exercised agency as a way of deliberately enacting their individual values, beliefs, and goals. The development of agency was described as a “complex continuous negotiation process between teachers’ personal characteristics, their sense of self (identity), and the context in which they work.”

In a recent study by Huang et al. (2021), data from three novice teachers in Hong Kong documented the dynamic changes of their English teaching beliefs and the impact of these changes on their identity construction and their professional agency. Findings indicate that the novice teachers’ belief is “variable, cumulative, and evolutionary,” and is influenced by the interaction between their individual meaning-making and identity-making, and the contextual realities (Huang et al., 2019, p. 202). Related to this, teachers may possess the capacity and are also ready for autonomy when situated in an invitational, supportive, and collaborative school environment (Huang et al., 2019). Whether teachers can become critically aware of the affordances (opportunities, possibilities, invitations, and enablements) in their working conditions determines whether they can exercise their teacher agency to act on these affordances to pursue their personal-professional development.

The studies presented above indicate that the development of teacher agency is imbued with affordances, challenges, and uncertainties in teachers’ working environments. Meanwhile, it is complex to underpin teachers’ decision to join, stay in, or leave the teaching profession. Individual teachers’ capacity in exercising agency may vary in different situations. The intensity of individual engagement with social suggestions may also vary, depending on individual interest and intentionality. Consequently, teachers’ professional agency may vary in its manifestations across individuals and situations and under different levels according to different aspects of a teacher’s professional work. It is thus essential to understand how teachers respond to contextual realities – opportunities and challenges – in exercising their professional agency in a longitudinal way. In this connection, it is fruitful to examine in-depth, teachers’ proactive, reactive, and passive agency during their early years of school teaching.

## Framework on Understanding Teacher Agency

**Figure 1:**
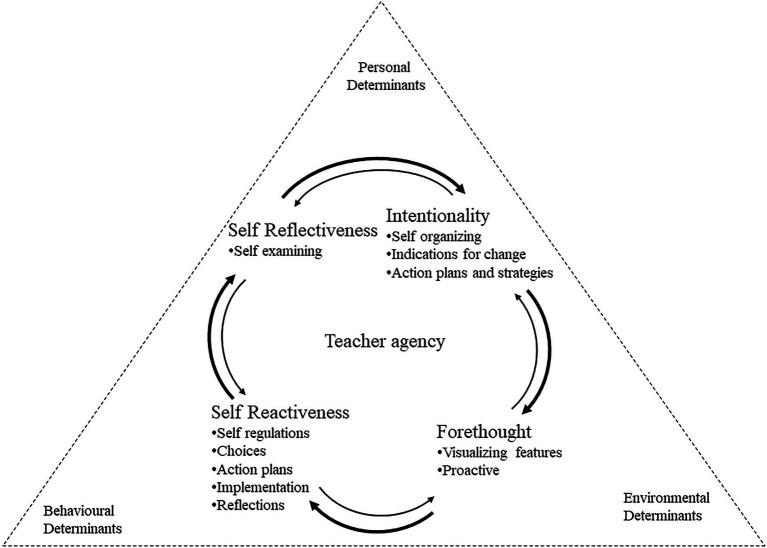
The Triadic Reciprocity Framework Core Agency Concepts (TRFCAC) model (Jenkins, 2020, p. 170).

This study analyzes data through the TRFCAC model (Figure 1) developed by Jenkins (2020). This framework was a combination of Model of Core Properties of Agency of Bandura (2001) and Triadic Reciprocal Causation Model of Bandura (1999). The framework enabled us to explore the complex dimensions of teacher agency, and the contextual factors that influence teachers’ actions.

In Bandura’s (2001) model, human agency possesses four core features i.e., intentionality, forethought, self-reactiveness, and self-reflectiveness. Intentionality is demonstrated when teachers intentionally plan for pedagogical change, as agency simply means “acts done intentionally” (Bandura, 2001, p. 6). Forethought refers to outcome expectations that can help one set long-term goals and anticipate problems. That is, “behavior is motivated and directed by projected goals and anticipated outcomes” (Bandura, 2001, p. 7). Self-reactiveness refers to an ability to give shape to appropriate courses of action and to motivate and regulate their execution. Self-reflectiveness refers to an ability to self-examine one’s metacognitive processing of thoughts, feelings, actions, and motivations.

Jenkins (2020) model also draws on causal model of triadic reciprocal causation of Bandura (1999), in which personal factors, behavioral patterns, and environmental events all operate as interacting determinants that influence one another bidirectionally. Personal determinants include personality, attributes, interests, skills, identity, affective states (moods), belief, and cognition. Behavioral determinants refer to one’s actions and practices in managing the change process through adapting behavior to a particular environment. Environmental determinants include the affordances and constraints inherent in the rapidly evolving recent and historical organizational environment. The focus on the three determinants is essential as learning occurs in the social context with a dynamic and reciprocal interaction among the person, behavior, and environment (Bandura, 2000). According to the description of human agency of Bandura (2000), people are partly the products of their environments, but by selecting, creating, and transforming their environmental circumstances they are also environment producers. Therefore, we see a need to apply the agentic theory in education as teaching practice is situated in an environment of goals and aspirations, outcome expectations, affective proclivities, impediments, and opportunities.

This study adopted the TRFCAC model to examine Hong Kong ESL teachers’ professional experiences in their early years of school teaching, with the following rationale. First, the four core features of personal agency, including intentionality, forethought, self-reactiveness, and self-reflectiveness (Bandura, 2001) are crucial in understanding teachers’ agentic actions. Second, the connection between the personal, behavioral, and environmental determinants in which teachers live and work may influence their early years of professional development.

Integrating two agency models of Bandura (1999, 2001), TRFCAC model of Jenkins (2020) helps explore early-career ESL teachers’ personal-professional pathways. The TRFCAC model reflects a need to explore “teachers’ sense of professional agency from a multifaceted perspective,” and consider their sense of agency at three levels, including the “individual, community, and organizational levels” (Eteläpelto et al., 2015, p. 664). There is thus a need to explore teacher agency based on the TRFCAC model, in particular, the dynamic interaction of intentionality, forethought, self-reflectiveness, and self-reactiveness on ESL teachers’ perception of personal, environmental, and behavioral determinants, to shed light on the challenges and constraints encountered by teachers in their work, and the resources accessible to them.

## Materials And Methods

### Research Questions

This study investigated the teaching lives of three ESL teachers, focusing on how they exercised their teacher agency to take control of their teaching and professional development at the early stage of their teaching career. The study was guided by the following research questions:

1. What beliefs did the ESL teachers uphold in the early years of their teaching career?
2. How did the ESL teachers exercise their teacher agency to take control over their teaching and professional development?

### Research Design, Participants, and Context

This study is primarily interpretative and qualitative in the sense that the same phenomenon can yield an array of interpretations from multiple perspectives by the research participants, researchers, or other observers. A generally qualitative design allows individuals to “construct reality in interaction with their social worlds” (Merriam, 2009, p. 22), which makes it possible for researchers to “develop an in-depth exploration of a central phenomenon” (Creswell, 2012, p. 206). Within this interpretative qualitative framework, the study adopted a longitudinal multiple-case study design to map three ESL teachers’ lived stories spanning more than 7years of their school teaching (August 2013–December 2020).

Kristy, Joyce, and Sammy were full-time ESL teachers in different secondary schools in Hong Kong. The anonymity of the participants and the schools where they were working was secured. These three female teachers were purposefully selected, meeting all the basic requirements, including teaching English in Hong Kong secondary schools for a few years upon graduation from a teacher education program, and being willing to share their life and teaching experiences with the researchers. This study also adopted a convenience sampling approach. All the three participants were former students of the first author. They were from the same cohort of a B.A. and B.Ed. English double degree program in a local university. They all began their school teaching after completing the English double degree program in summer 2013. More specific information about the participants is as follows.

Kristy returned to her alma mater to start her first year of school teaching upon graduation from the English double degree program. She left the school 3years later because she was not able to renew the contract. She then took up a teaching position in a directly subsidized school (DSS) in 2016. Although she encountered various difficulties in the DSS in the first year, she gained a sense of control over teaching in subsequent years. After finishing the fourth year in her second school, she had an opportunity to return to her alma mater to continue her teaching journey in the place where she started her teaching career.

Joyce also started teaching upon graduation from the double degree program. She left her first school after 1year of teaching due to geographical distance and perceived inadequate professional learning opportunities. She proceeded to another secondary school to continue her English teaching journey from September 2014 to August 2016. It was a Band One government school located closely to her home and she reported that she had a good time in this school. She left that school due to funding unavailability after 2years. In order to better prepare for her teaching career, she joined a 2-year part-time M.Ed. program, while taking a few temporary substitute-teacher positions in a few secondary schools, during 2016–2018. She went to the third school after completing the M.Ed. program. However, Joyce left the teaching position after 2months owing to the unfair treatment she received there. She later worked in an education-related NGO as an administrative officer for about half a year. She decided not to renew her contract and stayed home to take care of her young daughter, thinking about whether she would leave the teaching profession permanently.

Sammy taught English in a boys’ school upon graduation from the same double degree program. She spent 1year for her first teaching job, leaving that school in 2014 due to various challenges she encountered there. For instance, the overall atmosphere of a boys’ school did not suit her nature. After leaving her first school, she then enrolled in a 1-year full-time postgraduate diploma in psychology. She joined another school in 2015 after completing the program. During her service in the school, she pursued a 2-year part-time M.A. degree in comparative and literary studies. During the fourth year in her second school, she realized that she had hit a plateau in terms of her self-development. She began to search another job. Starting from September 2019, Sammy started teaching in her third school, a Band three school. She thought she could contribute more to teaching and student development than in her first and second schools.

### Data Collection

Aiming to reveal the complexities of teacher agency in the secondary school contexts in Hong Kong, the major source of data was in-depth interviews with three ESL teachers, Kristy, Joyce, and Sammy, to listen to their lived stories. Two main rounds of interviews were launched. The first round was conducted from 2013/2014 to 2014/2015, within the first 2 academic years of their in-service school teaching after graduation; and the second was conducted from 2018/2019 to 2019/2020, within their sixth and seventh/eighth academic years after surviving their initial years of school teaching. Each face-to-face interview lasted for more than 1h. The interviews were audio-recorded. English or Cantonese language was chosen for an interview when the interviewees felt more comfortable expressing ideas in English or Cantonese at that particular moment. All the interviews conducted in Cantonese were translated into English by a senior research assistant who is highly proficient in both Cantonese and English.

In addition to interviews, other data sources included personal communication with the participants and school documents from their workplaces, such as Schemes of Work and school timetables. Within the middle period (i.e., from their third to fifth academic year in in-service teaching), formal and informal conversations (*via* email/WhatsApp exchanges) with all the participants were maintained by the first author. All these supplementary forms of data other than in-depth interviews contributed to the understanding of each participant’s professional experiences in the Hong Kong educational settings.

### Data Analysis

The authors read the interview transcripts and other forms of qualitative data to familiarize themselves with the content and adopted thematic analysis to analyze the overall dataset (Merriam, 2009). Data analysis and interpretation occurred over multiple stages along with data collection and the move from field texts and research texts. Specifically, the process for data interpretation consisted of three main stages.

First, the researchers carefully coded the interview transcripts with particular attention paid to the constraints on and opportunities for teacher agency. This process was based on rigorous discussions and critical interpretations among the researchers.

Second, the constructed narratives, based on interviews and all supplementary data, were shared with the teacher participants for further data additions, amendments, and clarifications. Such approach not only ensured the trustworthiness of the data analysis, but also elicited more information through the participants’ further elaborations of stories (Barkhuizen et al., 2014).

Finally, after completing the first draft of research texts, the researchers re-examined the themes in depth through re-reading the original data and composing mini-stories with a focus on the specific time, space, and characters involved. We deconstructed, constructed, and reconstructed the social meanings through writing mini-stories that referenced the identified themes. The storyline of the three teachers’ narratives was then developed and knitted into “story constellations” (Craig, 2007).

## Findings

The following sections present the three ESL teachers’ teaching experiences in their initial 7years of school teaching. An examination of the stories they lived by will show the change of their professional agency over time.

### The Story of Kristy

Kristy who is approachable often shows her smiley face. She has been serious toward her studies, since her childhood. At the upper primary stage, she was placed in an elite class and her English teacher was an influential figure to her at that time. Kristy was influenced by the teacher, who emphasized knowledge and the learning process through interesting and innovative pedagogies. Her English learning in her junior secondary was considered the best experience because it was “more interesting and relaxing” (Interview, 31 August 2013). At university, she cherished the teaching practice incorporated into the overseas English immersion program at the end of the third academic year and also her two teaching practicums in local schools scheduled in the last 2years of the double degree curriculum. The school atmosphere nurtured her initiative to learn teaching. The positive experiences strengthened her determination to become an English teacher. She reported:

When I was in Primary 5 and 6, my English teacher provided me with some interesting and innovative ways for us to learn English. In secondary school, we had a good teacher model. We admired her and wanted to learn from her. After being admitted to the university education program, the teaching practice in the immersion program in Australia was really great. I was determined to be a teacher (Interview, 31 August 2013).

In short, Kristy’s personal experiences and feelings in becoming a teacher were built on her positive navigation during primary, secondary, and university education. The word “determined” indicates her goal to be a teacher.

#### First School (Alma Mater)

Having graduated from university, she started her teaching career on a contract basis in her alma mater. Kristy felt it grateful to work there despite some challenges. She attempted to implement activity-based teaching in her classroom through working hard to strike “a balance between the reality and ideal pedagogies learned in university” (Interview, 31 August 2013).

She was assigned two S2 (Secondary 2) classes and one S3 class in her first year. She faced a problem of learner diversity. Another difficulty was the curriculum. As she was new, she needed time to be familiar with the curriculum and design relevant materials to meet her students’ diverse needs. She also faced the problem of learners’ low English proficiency. She tried to orient her lessons to be task-based, and expected her students to be motivated. Her reported difficulties included the “lack of knowledge in dealing with weaker students,” and “arranging classroom activities” being “a luxury” in classroom teaching (Interview, 1 April 2014). The school explicitly emphasize both assessment for learning and assessment as learning. Kristy regarded such policy aligned with her teaching beliefs. However, during her first year of teaching, she reported she was rather “passive” in developing materials to meet students’ diverse needs and the school’s assessment requirements.

Her second and third years seemed easier. To help her better blend into this new working environment, her panel leader continued to arrange experienced teachers to guide her. With continuous support from colleagues, Kristy adapted better into the teaching environment from her second year. The initial experience and confidence obtained from her first year teaching, together with her perceived reduction of workload, enabled her to act more proactively in her own classroom. An informal interview in her third year of teaching was conducted when she paid a visit to the university, where she obtained her B.A. and B.Ed. double degree. She shared her experience with the first author (interviewer/discussant) highlighting proactivity in her second-third years, including “experimenting with task-based or activity-based methods” and “implementing assessment for/as learning” rather than “simply following assessment of learning” (Interview, 28 March 2016). She explained further in the interview:

I was able to adapt better into the environment from the second year. During the first year, it was indeed challenging to get hold of the curriculum…. In my second year, I was assigned two English classes only and the teaching materials for classes I taught in my first year could be used again…, and I also got four classes of Religious Studies, which did not add much workload.

We [Kristy and an experienced teacher] have been assigned to teach in the same form for 3years. There were a lot more chances for us to exchange ideas and insights mutually, rather than having her who simply told me what to do.

Overall, Kristy felt grateful in working in her alma mater. However, she had to leave the school unwillingly 3years later due to a reduction in the total number of classes which in turn affected staff funding.

#### Second School

Kristy then joined the DSS. She found it particularly difficult to adapt to the new teaching environment. She still felt the emotional attachment to her alma mater as she put, “I still missed the first school I worked in” (Interview, 15 April 2019). Worse still, the new workplace provided no structured support for her. As the school was relatively new with less than 20years of history, the teaching staff were quite young compared with those in her first school. Apart from the school system, students here were also different in terms of behaviors and characteristics. While her past students were quite conforming and easy to control, her new batch of students here were seen as “more challenging,” as they would “test the bottom line” of new teachers (Interview, 15 April 2019). Kristy reported that those students thought they were more familiar with the place than the new teachers. In terms of teaching content, although colleagues were quite open to discussions regarding the procedures and overall design of their lessons, they rarely shared teaching materials. Another challenge was lesson observation. As the school needed to have an External School Review (ESR) enacted by the Education Bureau, the administrators had an even more critical eye on the performance of individual teachers. She repeatedly told herself, “I am not the worst. I will not be fired” (Interview, 15 April 2019). She reported:

We needed to do ESR. I felt it not easy. There have been requirements on how to use different tools in implementing e-learning. The panel head filled in a lesson observation evaluation form and conducted an appraisal with me. The appraisal is related to the promotion… I wish I am not the worst (Interview, 15 April 2019).

Kristy found it difficult to adapt to the new teaching environment. She felt she was deprived of professional autonomy in teaching. She was stressful in handling the “top-down” appraisal and peer observation, for which, she had to strike “a balance between lesson preparation work, administrative work, and marking assignments” (Interview, 15 April 2019). She was thus thrown in bewilderment.

Nonetheless, Kristy’s positive beliefs (e.g., being helpful in student learning) motivated her to adapt to the new environment. After her first year in this school, she developed a better sense of the workplace and understood her own positioning as a teacher. She attempted to create more space for self-reflection and take greater control over teaching. She signed up for workshops and seminars provided by the Education Bureau to enhance her professionalism and to bridge the “theory-practice” gap (Interview, 18 December 2020). She endeavored to build a sense of efficacy and control as a teacher.

#### Coming Back to Her Alma Mater

After working for 4years in the DSS school, Kristy decided to return to her alma mater to start her eighth year of school teaching and she was successful in the end. She described the second school as “stressful” because teachers “have to take up responsibilities not only to the students but also to the parents” (Interview, 18 December 2020). She described some of the students as “spoiled.” Their mindset was “why the teacher cannot make me understand the knowledge” rather than “please teach me about this. I do not understand it” because they were “the one who pays the tuition” (Interview, 18 December 2020). In contrast, she descried her enjoyment as a teacher after returning to her alma mater as the students were more motivated.

Here, the students will ask as many questions as they can. They will ask you if you can mark extra writing too. And that brings you a bit of pressure. It is a pleasure to see students’ growth (Interview, 18 December 2020).

It appears that students’ diligence and desire for knowledge is a source of stimulus for Kristy’s teacher agency development. In addition, the school system is an important factor. According to Kristy, although the previous DSS school seemed to allow more flexibilities in teaching, there was an obvious insufficiency of specific guidelines. In her alma mater, the teaching schedule was tighter, but the school provided more ready-made resources, specific guidelines, and support from senior teachers. These were important for busy teachers in Hong Kong, as described by Kristy.

Continuing her teaching journey in her alma mater, Kristy started to highlight the importance of “work-life balance.” She expressed that she could set a roadmap that could relieve her stress in her alma mater. In her views, “work is not the only thing” in her life, though working is important. She mentioned that work-life balance made her feel “being integrated to the working environment” (Interview, 18 December 2020).

### The Story of Joyce

Joyce has a cheerful personality. She studied in a primary school where English teaching was, in her words, “teacher-centered.” She completed her secondary education in a prestigious girls’ school, where the teachers adopted a more naturalistic approach to language teaching and created a less structured and more relaxing environment for language learning. After the A-level examinations, she decided to enroll in a teacher education program in a local university mainly because of the teachers she met. The idea of being a teacher budded when she was in primary school. It got shaped and reinforced throughout her secondary education. As she often found joy in teaching, she embarked on her teaching career after graduation. In an interview at the very beginning of her first year of school teaching, she clearly explained her passion in teaching:

Being a teacher is very good because we are helping the kids grow. It is an honor to be a teacher. As I found joy in teaching others, I embarked on my teaching career right after graduation. I just wanted to be a teacher (Interview, 31 August 2013).

This quote indicated being a teacher was her desired identity. Although Joyce was optimistic toward her first teaching job, her career path turned out to be the least stable among the three teacher participants.

#### First School

Joyce started her first teaching job at a local secondary school in summer 2013. When asked whether she would be confident to find her place at the school, she highlighted the heavy administrative workload. Like other novice teachers, she was assigned a mentor-teacher who would chat with her about life and teaching affairs. She also benefited from the teaching materials shared by other teachers. In her students’ eyes, she was not a strict teacher, but she communicated expectations and rules very clearly with students at the beginning of the school year. As time elapsed, she was like a friend to her students. It was her philosophy that the teacher does not just impart knowledge but also life values. This “teaching-beyond-schooling” approach was in line with her broader belief that it is “an honor to help somebody grow.”

In summary, Joyce enjoyed the initial months of her teaching. After the first half of the academic year, her self-doubt grew, as she remarked (Interview, 25 January 2014):

When I do not find job satisfaction sometimes, I think whether I am suitable in this job and sometimes I wonder if I should ever be a teacher. Should I just leave the school and the whole industry completely because if I am not doing a good job, I am not only affecting myself, I am affecting the others as well…

I do not feel being appreciated [by students]… [I get] not really positive comments from colleagues…

Her perceived mediocre job satisfaction and her self-doubt as quoted above stemmed from several classroom management challenges, a couple of complaints from her students and a lack of appreciation from her colleagues. The lack of appreciation might partly explain why she chose not to renew her 1-year contract. Two other factors also contributed to this decision. First, the geographical distance took her one and a half hours to travel to work. Another crucial factor contributing to her quitting despite the gloomy job market at that time was her desire to work with people whom she could feel more connected to. The school was a young school with young teachers and administrators who did not bond well with her. Some colleagues may compare themselves with her as she explained below some years later:

I did not feel the sense of connection even though we were not enemies. I would really love to collaborate with and learn from teachers from different backgrounds and of different ages. However, since other teachers and I are more or less the same age, our mentality and thoughts were too similar as well…. Also, there were comparisons among teachers. When all the teachers are almost at the same age, students tended to compare. Anyway, that was not the working atmosphere that I prefer (Interview, 6 April 2019).

#### Second School

Continuing her teaching journey, Joyce joined another secondary school, which was a Band One government school in closer proximity to her home. The position was based on a 2-year contract. She was responsible for teaching senior form English and this gave her some pressure. In addition, the school required lessons in the same form to cover exactly the same content, which constituted a particular difficulty for teachers like Joyce who wanted to develop materials and strategies to meet students’ different needs. As a result, she found her first year there “unsatisfactory” and described it as “a period of exploration” (Interview, 6 April 2019).

Fortunately, her colleagues shared materials among teachers and were eager to collaborate and exchange pedagogical ideas, during lunch or after school. This motivated Joyce to improve her teaching. She thought that she did not actually know her students well enough; she held a firm belief that “taking students’ learning needs into consideration is of primary importance” (Interview, 6 April 2019). She then started to collect students’ feedback on her teaching through casual chats with students after class and giving out a piece of paper to collect feedback.

In her second year, she adjusted her teaching with reference to students’ feedback. Although her teaching was guided heavily by the Scheme of Work, she did not find it constraining and was able to create space for manouevre in delivering the content (e.g., prioritizing the teaching of generic reading skills, which would empower her students to come up with correct answers on their own). Her 2 years at this school concluded in joy and satisfaction, leading to a higher level of teacher agency.

After working in two schools for consecutively 3years, she desired a break from full-time engagements and turned to a part-time 2-year (2016–2018) M.Ed. program.

#### Third School (September–November 2018)

The M.Ed. studies with intermittent breaks also indicate Joyce’s turning point. She decided to devote more time to nurturing her young daughter. When asked whether she was considering a full-time teaching job after completing her M.Ed. studies, she said it was “a possible option” (Interview, 6 April 2019), although the (third) school she served did not give her a pleasant experience. She was only informed of the classes that she was teaching a few days before teaching was meant to start. She received little concrete support from the department and colleagues and there was no recycling of shared teaching materials. Moreover, surveillance mechanisms, like homework inspection and lesson observation, were implemented as part of the “quality control” measures in the school. While these measures may be common in Hong Kong nowadays, “this school would do it repeatedly until the ‘bosses’ felt satisfied.” She reported further:

I have never experienced such a lesson observation. Some teachers observed my lessons and commented that I could not live up to their standard. The principal stood at the door of my classroom for a long period without leaving, and I felt offended. I was also offended by the fact that a Chinese history teacher was there to evaluate my teaching and I do not see why this Chinese history teacher was qualified to judge how I teach English. I felt untrusted and disrespected… (Interview, 6 April 2019).

The observers criticized Joyce’s performance. Joyce described the criticism as “denouncing opponents during the Cultural Revolution” in China. A second lesson observation was scheduled and Joyce handed in her resignation next day to quit the job she did for only 2 months. The experience was traumatic, making her consider “whether I am still suitable to be a teacher or not.” It took her at least 1 whole month to restore a mental balance and she said that she still found herself “mentally and spiritually vulnerable” half year after the lesson observation (Interview, 6 Apr 2019).

#### Stay in or Leave the Teaching Profession?

Joyce had doubt over her teaching capability and fear of repeating the traumatic episode on the teaching frontline. She later resumed teaching as a cover teacher in a school during January–July 2019, and worked as an education administrative officer in an NGO during August–December 2019. The administrative experience provided her a chance to compare the nature of school teaching and administrative work. Given a choice, she would prefer to be a teacher as demonstrated by what she explained in several interviews during 2019–2020: “Being a teacher is never just because of the salary”; “I see teaching as everyday creativity”; “As a teacher, you can develop a rapport with your students.” However, at this point, she prioritizes her family commitments and being the teacher on a long-term basis became unrealistic.

After a year of struggle due to the need to take care of her daughter during the COVID-19 pandemic and the “suppressing political environment in Hong Kong” since 2019, she finally decided to “leave Hong Kong for her daughter’s future education” (Interview, 7 July 2020). She continuously questioned her teacher identity because “there were incidents regarding teachers’ so-called professional conduct in the educational field” (Interview, 18 November 2020).

### The Story of Sammy

Sammy is a cheerful girl. With her sincere attitude, she befriended a lot of like-minded people in the university. She did not expect to be a teacher. She regarded the education component of her English double degree would afford “more chances to interact with people, like during my teaching practices.” Throughout the university education, she kept reflecting on the question, “Am I suitable to be a teacher?”

During the 4years of university study, I reflected on myself how to make a change for my life. I have never thought of being the teacher before entering university. Am I suitable for being a teacher? (Interview, 31 August 2013).

However, Sammy was determined to become the teacher upon graduation because she considered herself as an agent of pedagogical innovation who would definitely bring changes to the educational landscape.

#### First School and Postgraduate Diploma in Psychology

After weighing different offers upon graduation, she finally settled into a Chinese as medium instruction (CMI) boys’ school. She set out with an approach described as “half-new-half-old” in her own words (Interview, 31 August 2013). She preferred traditional direct instruction and drilling for grammar and vocabulary especially for weaker students; meanwhile, she strived to “create space for interactive classroom activities with an emphasis on the practical use of English in a fun and authentic way.” She believed that her role was to facilitate her students’ learning to the degree that they were able to “use English to communicate and interact with people, to love the other countries, to love the cultures, to love humanity” (Interview, 16 January 2014).

In her first year, she was responsible for one S3 class and two S1 classes, on top of her duty to run the English Corner, to organize a joint-department competition and to be a Parent-Teacher Association member. Her mentor gave her much advice but other colleagues were too busy to help her. Like many beginning teachers, she struggled to prepare materials. Another difficulty she encountered was the group of students with special education needs (SEN).

My class was mostly SEN students. I lacked knowledge in dealing with SEN students. I tried to incorporate interactive activities to make English teaching fun, but such efforts were in vain (Interview, 16 January 2014).

It appears that dealing with SEN students and preparing teaching materials mainly contributed to her hectic life as a beginning teacher. This compromised her original work-life balance. She was generally not satisfied with the fact that she “could only fulfill the assigned job duties with limited creativity and innovation” (Interview, 16 January 2014).

At the end of the school year, she decided to leave this school for three reasons: first, the overall atmosphere of a boys’ school did not suit her nature; second, she was only assigned junior forms in her first year and this might weaken her competitiveness as a capable secondary school teacher; third, she was responsible for helping coordinate the Parent-Teacher Association and this duty was not what she desired. Then she started a postgraduate diploma (psychology) study in a local university, hoping to get inspired by seeing matters with new perspectives. Satisfactory completion of this program also promised alternative career pathways like a counselor and an educational psychologist. At that point, she still thought that being a teacher allowed her to design powerful and innovative learning environments for her students.

#### Second School and M.A. Studies

After a 1-year study break, she continued her role as a teacher in the DSS. She perceived more room for manoeuvre here as there was a core central curriculum. She had freedom to interpret the curriculum and teach in the ways she considered appropriate and effective. She saw more learner diversity accentuated by some SEN students. The relationships among colleagues were not bad; she had the best relationships with her panel head and peers of her age range. Her first year at this school was not too difficult, although a considerable amount of time was spent exploring the school policies and culture.

I managed to negotiate for a change in curriculum to enhance teaching and learning. I received professional support from my panel head and my teacher fellows. I was able to bring in new ideas for my students’ various needs. There were more flexibilities than in my first school (Interview, 31 March 2019).

She obtained her first opportunity to teach a senior class. She was eager to teach the class and witness the students graduate. Her school did not usually allow teachers to take courses within school hours. She took advantage of her after-school time to take courses or seminars organized by the Education Bureau and British Council to keep herself abreast of the latest pedagogical knowledge. Another way she adopted to empower her teaching was that she often exchanged ideas with her university classmates. She was able to pick up various teaching strategies as her classmates were teaching at different schools.

From the start of her third year, she started to feel teaching duties were the same and there was much less she could learn from her colleagues, as sharing was not a norm in the department. She only managed to talk with her peers for new ideas and comments. In spite of this, her strong belief led her to pursue her second higher qualification, M.A. in comparative and literary studies, in the same university where she obtained her postgraduate diploma: “If I just do things to satisfy the basic requirement, I will not be able to improve my teaching” (Interview, 31 Mar 2019). This degree did not bring explicit impacts on her teaching, as this school did not stress the acquisition of literary knowledge. Nonetheless, she gained the necessary knowledge and tools to critique various literary works. The professors were mostly inspiring and taught the courses through classroom activities. This activity-based approach combining with a problem-solution model derived from her previous psychology background formed her teaching style. Whenever she saw a problem arising from her class, she would start researching and evaluating ways to solve the problem.

The fourth year at this school was not too easy as she realized that she had hit a plateau in self-development. While expecting to be newlywed toward the end of that year, she felt more intensely about the plateau and stagnation she faced in her job. With increasing administrative tasks, she felt “a sense of powerlessness”; moreover, she acknowledged that this situation is “not healthy” and that it depended very much on her “own initiative to sustain professional growth” (Interview, 31 Mar 2019), as she thought there was limited new pedagogical input and discoveries for her. As a capable teacher, the school tended to assign her more duties and responsibilities to the extent that she felt the workload was “saturated” (Interview, 22 Aug 2019). Feeling confused, she started to send cover letters to other schools to see how much she was treasured from other schools’ perspectives.

#### Third School

After rounds of interviews, she finally secured an offer from a lower-achieving CMI school. This school expected her to take up more functional roles. The school had a strong emphasis on students’ mental well-being and self-management, providing her with opportunities to draw on her counseling expertise to support students. Since the students’ English proficiency was generally low, she spent more time scaffolding their learning process and employing activity-based approach in teaching. The students began to like her and some sent cards to show appreciation.

In her second year, catering for her students’ diverse abilities was still challenging for Sammy. Fortunately, she benefited greatly from the sharing culture, which in turn informed her material preparation and lesson planning. She explained how “by far the best sharing practice here” and the reflective atmosphere would foster her professional growth:

… Almost every teacher is willing to share. I think that my colleagues are very reflective. Since they prepare a lot of resources for their own classes, and if they consider resources prepared by them useful for other colleagues. They will share for sure (Interview, 10 Dec 2020).

Another good practice was lesson observations:

… Lesson observations are absolutely professional. Teachers or supervisors discuss together after observing a lesson. Observing teachers have a pre-meeting with the panel head before sitting lessons. …Although I have experienced many lesson observations in the past…, the practice here is still an in-depth learning process to me (Interview, 10 December 2020).

This presented a stark contrast to lesson observations as a routine administrative measure almost devoid of meaning in many other schools. In addition to lesson observations, there were many other professional development opportunities for English teachers. For example, the school organized 5–6 professional sharing sessions about what individual teachers have learned in their professional-development courses each year. The activities aimed to disseminate good practices within the English department.

Interestingly, Sammy became more willing to take on non-academic duties after joining this school. She requested to join the careers team to which she thought she could contribute more. She did not confine herself in her comfort zone and had opened up herself to challenges and opportunities. She also became more confident in teaching and demonstrated a sense of self-actualization through capitalizing on her strength.

In summary, Sammy has been engaging more in reflective teaching and pedagogical innovations since joining her third school. When asked whether this school was for her long-term development, she steered away from the focus to a more philosophical argument and subtly dismissed this question as unimportant:

What is more important is to go with the flow as everywhere has its own constraints and opportunities. It is never-ending to be on the quest of being a teacher. So long as you know your place, you can be a good teacher (Interview, 10 December 2020).

This zen-like mentality is definitely an inspiring one that blends well with her own philosophies and her other aspects of life. Within 2 years of teaching here, she has definitely enhanced her sense of agency and her capacity to take control over teaching and personal-professional development.

## Discussion

**Figure 2:**
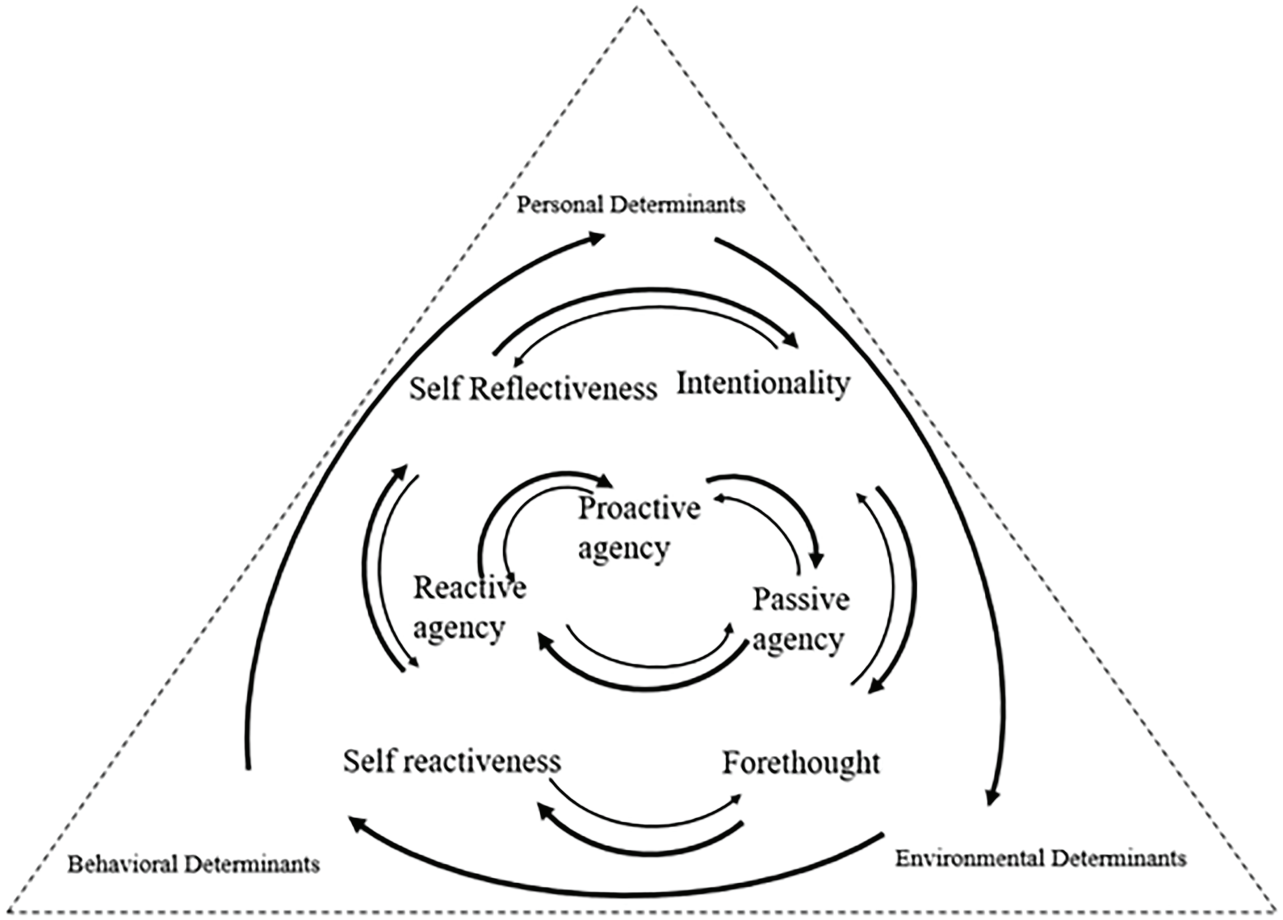
A proposed three-layered Triadic Reciprocity Framework on teacher agency (based on Jenkins, 2020, p. 170).

The findings document how three Hong Kong secondary ESL teachers exercised their teacher agency to construct their desired professional identities at the early stage of their school teaching. Based on the findings, this study proposes a three-layered triadic reciprocity framework on delineating the complex process of teacher agency development (Figure 2). The three-layered triadic reciprocity framework extends what our knowledge about the TRFCAC model (Figure 1).

### The First Layer: Proactive, Reactive, and Passive Agency

In the first inner layer, the three teachers demonstrated different degrees of proactive, reactive, and passive agency. Proactive agency enabled teachers to obtain a sense of ownership, and possibly, “goal-directed, effortful, and proactive engagement in work practices” (Goller, 2017, p. 20). Proactive agency was motivated by a number of determinants such as collegial support and mentoring. Reactive agency occurred as a result of forced or required changes in teaching. Passive agency occurred when teachers have low efficacy and little motivation to enact changes. For example, while Kristy held a strong belief for interactive and innovative teaching, she displayed passivity in designing materials to meet her students’ diverse needs in the first year in her first school (alma mater). When coming to the second and third years in her alma mater, she demonstrated proactive agency in innovating classroom teaching and assessments partly due to receiving adequate support from senior teachers. After moving to her second school, the appraisal system brought stress to her classroom teaching. She merely demonstrated reactive agency in accomplishing her teaching assignments, and thus, she missed and eventually sought a chance to return to her first school, her alma mater, in which she was afforded more opportunities to exercise her teacher agency (and thus teaching was more manageable) and in which her professional autonomy was less constrained than in her second school.

In the case of Joyce, her positive feelings toward future teaching career determined her attitude in pursuing a strong sense of teacher agency. When encountering obstacles and setbacks (e.g., underappreciation from both her students and colleagues), and when perceiving inadequate connection to the teaching community and collaboration among colleagues, in her first school, she merely wanted to fulfill the school requirements reactively. However, she exhibited proactive agency in teaching innovation in her second school, afforded by full and friendly interaction with colleagues, adequate collaboration among colleagues (e.g., shared materials in the English department), and the establishment of a good rapport with her students. After 2 months of teaching in her third school, she resigned her full-time teaching position triggered by, in her own words, an “insulting” and “Cultural Revolution” alike lesson observation, and the perceived authoritarian school culture (a lack of professional autonomy). The critical experience in her third school made her become passive in enacting professional agency. She needed to make a difficult choice between leaving the teaching profession permanently and resuming teaching to continuously “see kids [her students] grow” (her own words in several interviews after leaving her third school).

In the case of Sammy, feeling confident upon entering the teaching profession and considering herself as an agent of educational innovation, she took proactive agency in her own classroom at the beginning of her first year in her first school through a mixed mode of what she termed “traditional teaching” (direct instruction and drilling for grammar and vocabulary especially for weaker students) and communicative teaching (organizing interactive activities and emphasizing the practical use of English in a fun and authentic way). Given the perceived various contextual constraints in her first school, she demonstrated reactive agency in seeking necessary pedagogical implementation to simply meet learners’ diverse needs (that is, as what she sometimes remarked, “to get the job done as you are, after all, paid”). In the first 2years in her second school, she was able to exercise proactive agency (e.g., seeking various opportunities to enhance her professional competence by attending courses or seminars in her spare time), when she was given the first opportunity to teach a senior class (which she could not have in her first school), when she was granted the freedom to interpret the curriculum, and when she had successfully built good relationships with her colleagues. However, after working for 3 or 4years in this school, she became passive in taking actions to improve teaching because she realized that she had hit a plateau in terms of professional development. She demonstrated proactive agency in her third school again because she found the real meaning of being a teacher from students’ recognition and respect and from colleagues’ genuine sharing of new ideas and classroom practices. Overall, Sammy’s experiences in the three schools tended to reveal that teacher agency is dynamic and fluctuating and thus mirrors a close relationship between self and others, and between self and contexts.

### The Second Layer: Self-Reflectiveness, Intentionality, Forethought, and Self-Reactiveness

In the second layer, the three ESL teachers’ attempts to exercise proactive, reactive, and passive agency were influenced by the four properties of human agency, i.e., intentionality, forethought, self-reactiveness, and self-reflectiveness (Bandura, 2006). As argued by Bandura (2006, p. 164), teachers can also be “self-organizing, proactive, self-regulating, and self-reflecting.” In the case of Sammy, she demonstrated a need to form intentionality to change teaching practices and work toward realizing these plans, since she joined the teaching profession. Sammy believed her role was to facilitate her students’ learning to the degree that they were able to use English to communicate. She formed a forethought with which she was able to see successful future and work for it. Her forethought included the ability to use anticipated outcomes to guide and influence current activities and to promote self-reflective and foresightful behavior, which contributed to her agency development in the long term. Compared with Joyce and Kristy, Sammy could better adapt her actions toward the planned goals and changes through carefully monitoring and regulating her classroom innovations over the years (evidence of self-reactiveness). Sammy kept reflecting on how her “teaching can help the students” since her first year of in-service teaching. Such self-reflectiveness kept motivating her to critically self-examine her actions and continuously innovate her classroom practices, e.g., making choices, planning actions, maintaining motivation, monitoring implementation, and making adjustments when appropriate.

Kristy’s outcome expectations (forethought) originated from her positive navigation of experiences during primary, secondary, and university education. She formed intentions to orient her innovative lessons, and hoped her students could be motivated in learning English in her first school (alma mater) and second school. However, the “top-down” appraisals and peer classroom observation in her second school deprived her positive reactions and self-reflection in teaching.

Joyce was frustrated in her first school, due to inadequate collaboration between teachers and a disconnection to the teaching community. This affected her forethought for teaching and professional development. Sammy was disempowered by having little knowledge of teaching practices for SEN students until she started to really benefit from her colleagues’ genuine sharing of materials and ideas for teaching SEN students in her third school. The institutional structures of Sammy’s second school also suppressed her self-reflection, for which, she felt intense about the plateau and stagnation in teacher development. Sammy thus perceived passive agency in innovating teaching in her second school.

Overall, tensions and constraints in the teaching community impacted the three teachers’ intentionality, forethought, self-reactiveness, and self-reflectiveness, for which they may not adopt attitudes “that will sustain them in teaching” (Schaefer and Clandinin, 2011, p. 291). Factors, including classroom pedagogy, school administration, inexperience, insufficient knowledge, student behavior management, and even their job satisfaction, influenced the three teachers’ intentionality, forethought, self-reactiveness, and self-reflectiveness. Perceptions of teacher agency can significantly change over time, most notably in intentionality and forethought. Such changes influenced the three teacher’ self-reflectiveness and self-reactiveness.

### The Third Layer: Personal, Environmental, and Behavioral Determinants

In the third layer, personal, environmental, and behavioral determinants functioned together and influenced the three teachers’ professional agency. The mismatch between the perceived and actual practices of teaching was a constraint to the teachers’ awareness of actions for exercising agency in teaching. The three teacher participants daily teaching routine was inhibited by prevailing institutional expectations and standards of what they should achieve in their profession. For example, Joyce’s second school required lessons in the same form to cover exactly the same content. This environmental determinant may lead to the fact that Joyce found it challenging to address students’ various needs. In Kristy’s case, the ESR in her second school exerted pressure for her to strike a balance between reality and imagination and between administrative and teaching work. Kristy found it challenging to “negotiate multiple demands within schools and school districts” (Schaefer and Clandinin, 2019, p. 59). In Sammy’s case, the workload and degree of responsibility was almost the same in the three schools she had served, but stress and tension were remedied by the school’s assistance that enables her to initiate agentive work in teaching.

The current research highlighted how personal determinants were influenced by environmental and behavioral determinants. One incidence was that Joyce felt particularly “offended” in her third school, as a Chinese history teacher evaluated and criticized her teaching. The battle for place and presence in a school curriculum had weakened her willingness to exercise agency. Sammy felt unsatisfied with difficulties in teaching SEN students due to the lack of relevant psychological knowledge and with the assigned duties of leading the Parent-Teacher Association in her first school. Kristy’s extra burden was brought by ESR, inadequate colleagues’ support, and demotivation of students in her second school. The imbalance in work influenced her “exploration of new possibilities for action and new assumptions about context, self and practice” (Garner and Kaplan, 2019, p. 28). Teacher agency was thus constrained when a repository set of unauthorized and sometimes unacceptable values implicit to a school were at odds with values held by the teachers.

## Conclusion

This study examines the teaching lives of three secondary school ESL teachers in Hong Kong. The findings uncovered struggles that impeded their exercise and development of teacher agency. The findings provide insights into emerging ESL teachers’ personal and professional development. The discussions on teacher agency development point toward a three-layered Triadic Reciprocity Framework in explaining ESL teachers’ agency. The framework sheds light on the enactment of agency, which is context-specific. The three teacher participants experienced high agency in one area and low agency in another. The extent to which agency was exercised by the teachers depended on their positions within their collective organizations that were characterized by institutional cultures, such as administrative style, working atmosphere, special policies, and peers’ attributes.

We acknowledge some limitations of our inquiry, for instance, a small sample size in the Hong Kong context. However, this in-depth study based on different data sources over 7–8years portrayed a fuller picture of teacher agency. We conclude with pedagogical implications based on the findings.

First, system-wide structures may hinder teachers’ capacity in taking control of their teaching and personal-professional development. The subtle, yet sensitive power relation between teachers and school stakeholders can lead to teachers’ tensions regarding classroom authority and their confusion regarding their status as a teacher. It is thus essential for policy makers to attend to teachers’ needs in autonomous teaching, and to provide early career teachers with the time to develop their teacher identities rather than expecting them to cope with the work normally reserved for more experienced teachers.

Second, the findings suggest a possibility for creating conditions for teachers to take initiatives in constructing, generating, and extending their knowledge in teaching. Teachers need support to sustain their development and construct their professional identities. One worthwhile pursuit is guiding teachers to instigate deeper reflection on being and becoming the teacher. The early career teachers should be reminded to attend to the aspects that may not be readily apparent to them during their initial years. Finally, a suppressive environment may result in a lack of teacher agency, which may also give rise to the chronic problem of teacher attrition among early career ESL teachers. Teachers should reflect on the working conditions that affect their teacher autonomy, their beliefs and motivation in adopting agentic behaviors, and their professional identity development (Benson and Huang, 2008).

## Data Availability Statement

The original contributions presented in the study are included in the article/Supplementary Material. Further inquiries can be directed to the corresponding author.

## Ethics Statement

The studies involving human participants were reviewed and approved by Hong Kong Baptist University Committee on the Use of Human and Animal Subjects in Teaching and Research (HASC). The patients/participants provided their written informed consent to participate in this study. Written informed consent was obtained from the individual(s) for the publication of any potentially identifiable images or data included in this article.

## Author Contributions

JH led the research project and contributed to the research design, data collection, data analysis, article drafting, and reviewing and revising. JY analyzed the data and reviewed and revised the manuscript. All authors contributed to the article and approved the submitted version.

## Funding

This work was supported by the Research Grants Council of the Hong Kong Special Administrative Region, China (RGC Ref No. 12607418).

## Conflict of Interest

The authors declare that the research was conducted in the absence of any commercial or financial relationships that could be construed as a potential conflict of interest.

## Publisher’s Note

All claims expressed in this article are solely those of the authors and do not necessarily represent those of their affiliated organizations, or those of the publisher, the editors and the reviewers. Any product that may be evaluated in this article, or claim that may be made by its manufacturer, is not guaranteed or endorsed by the publisher.
